# Can we prepare healthcare professionals and students for involvement in stressful healthcare events? A mixed-methods evaluation of a resilience training intervention

**DOI:** 10.1186/s12913-020-05948-2

**Published:** 2020-11-27

**Authors:** Judith Johnson, Ruth Simms-Ellis, Gillian Janes, Thomas Mills, Luke Budworth, Lauren Atkinson, Reema Harrison

**Affiliations:** 1 School of Psychology, University of Leeds, Leeds, LS29JT UK; 2 Bradford Institute for Health Research, Bradford Royal Infirmary, Bradford, BD96RJ UK; 3 School of Public Health and Community Medicine, University of New South Wales, Sydney, 2052 Australia

**Keywords:** Resilience, Resilience coaching, Occupational stress, Healthcare workforce, Adverse events

## Abstract

**Background:**

Healthcare professionals are experiencing unprecedented levels of occupational stress and burnout. Higher stress and burnout in health professionals is linked with the delivery of poorer quality, less safe patient care across healthcare settings. In order to understand how we can better support healthcare professionals in the workplace, this study evaluated a tailored resilience coaching intervention comprising a workshop and one-to-one coaching session addressing the intrinsic challenges of healthcare work in health professionals and students.

**Methods:**

The evaluation used an uncontrolled before-and-after design with four data-collection time points: baseline (T1); after the workshop (T2); after the coaching session (T3) and four-to-six weeks post-baseline (T4). Quantitative outcome measures were Confidence in Coping with Adverse Events (‘Confidence’), a Knowledge assessment (‘Knowledge’) and Resilience. At T4, qualitative interviews were also conducted with a subset of participants exploring participant experiences and perceptions of the intervention.

**Results:**

We recruited 66 participants, retaining 62 (93.9%) at T2, 47 (71.2%) at T3, and 33 (50%) at T4. Compared with baseline, Confidence was significantly higher post-intervention: T2 (unadj. *β =* 2.43, 95% CI 2.08–2.79, *d* = 1.55, *p* < .001), T3 (unadj. *β =* 2.81, 95% CI 2.42–3.21, *d* = 1.71, *p* < .001) and T4 (unadj. *β =* 2.75, 95% CI 2.31–3.19, *d* = 1.52, *p* < .001). Knowledge increased significantly post-intervention (T2 unadj. *β =* 1.14, 95% CI 0.82–1.46, *d* = 0.86, *p* < .001). Compared with baseline, resilience was also higher post-intervention (T3 unadj. *β =* 2.77, 95% CI 1.82–3.73, *d* = 0.90, *p* < .001 and T4 unadj. *β =* 2.54, 95% CI 1.45–3.62, *d* = 0.65, *p* < .001). The qualitative findings identified four themes. The first addressed the ‘tension between mandatory and voluntary delivery’, suggesting that resilience is a mandatory skillset but it may not be effective to make the training a mandatory requirement. The second, the ‘importance of experience and reference points for learning’, suggested the intervention was more appropriate for qualified staff than students. The third suggested participants valued the ‘peer learning and engagement’ they gained in the interactive group workshop. The fourth, ‘opportunities to tailor learning’, suggested the coaching session was an opportunity to personalise the workshop material.

**Conclusions:**

We found preliminary evidence that the intervention was well received and effective, but further research using a randomised controlled design will be necessary to confirm this.

**Supplementary Information:**

The online version contains supplementary material available at 10.1186/s12913-020-05948-2.

## Background

Healthcare professionals (HCPs) are currently facing unprecedented pressure which has been exacerbated by the Covid-19 crisis. However, even prior to this crisis, staff were reporting record levels of stress and burnout. In the UK, the proportion of National Health Service (NHS) staff feeling unwell due to work-related stress increased from 28% in 2009 to 40% in 2018 [1] and in a recent study of seven European nations, it was estimated that around a third of doctors and nurses were suffering from burnout [2]. In the US this figure is even higher, with over half of physicians meeting criteria for burnout [3]. In addition to impacting workers’ personal wellbeing, HCP stress and stress-related sickness influences patient care, with staff stress and burnout consistently linked with the delivery of poorer quality, less safe care [4]. However, while this problem is now well recognised, identifying targets for interventions to reduce stress and burnout has been challenging.

Broadly, there are two approaches to reducing occupational stress; 1) reducing work demands, and 2) increasing workplace support [5]. Interventions which focus on the former are a type of ‘organisation-directed’ intervention as they aim to change workplace factors which create stress, and these have often been promoted as the most desirable form of stress-reduction intervention [6]. In contrast, workplace support interventions aim to enhance staff wellbeing by offering stress-management interventions such as training, counselling or assistance programmes. While these have been the most researched type of intervention for reducing burnout in healthcare professionals [7], they have been criticised for failing to address the real causes of stress.

While organisation-directed interventions which aim to tackle the causes of HCP stress are unarguably important, the fact remains that healthcare occupations involve intrinsic challenges which cannot entirely be removed. These include involvement in adverse events, where an error in patient care delivery results in patient harm. Such events arise in 10% of hospital admissions [8] and persist despite 20 years of investment in reducing their occurrence [9–11]. In addition to the harm and distress these events cause patients and their families, they can be extremely stressful for HCPs who often experience anxiety, depression and even symptoms of post-traumatic stress as a result [12]. Other stressful events which are intrinsic to healthcare work involve the communication of bad news to patients [13], managing sudden patient deaths [14] and treating distressed or aggressive patients [15]. Such events will increase with Covid-19 pressures, leading to a rise in the number of critical patients and unexpected deaths occurring and may increase the rate of patient safety incidents. In addition to creating stress in themselves, these events exacerbate the ongoing intrinsic demands of healthcare work, such as the management of complex patients [16] and the emotional labour of caring [17]. For these stressful clinical events and demands made of staff, workplace support interventions are crucial [6].

Resilience interventions are one type of workplace support intervention. ‘Resilience’ describes an individual’s capacity to maintain emotional equilibrium in response to difficult experiences [18] and the value of resilience for health is widely acknowledged [19–21]. Resilience interventions pro-actively develop the psychological skills which contribute to resilience, and several previous studies indicate these may confer a range of benefits on healthcare professionals, including lower levels of depression and burnout and increased well-being [22–24]. However, resilience interventions have been particularly contentious in healthcare, as they have been viewed by some as vehicles which can benefit organisations to the detriment of individuals; compensating for organisational problems by enhancing employees’ ‘hardiness’ [25, 26]. The negative perception of resilience interventions has been compounded by their broad focus of creating general coping skillsets; these are often taken ‘off-the-shelf’ and are not focused on addressing the intrinsic and unique workplace stressors that specific groups of HCPs face in their work.

Resilience interventions cannot compensate for needed organisational changes, yet a tailored intervention could address the intrinsic demands of healthcare work which cannot be addressed by other means. At present, no resilience intervention which is focused specifically on the intrinsic demands of healthcare work has been tested and only a limited number of studies have evaluated any type of resilience intervention in healthcare employees [27, 28]. Furthermore, those which have been conducted have usually focused on single disciplinary professional groups (e.g., [22–24]). In order to address this, we developed and evaluated a resilience coaching intervention to prepare healthcare professionals for the occurrence of stressful healthcare events, particularly adverse events. We focused on adverse events because while they are known to be particularly stressful for healthcare professionals, no intervention of any type which pro-actively supports healthcare professionals for these events has been evaluated. Peer support programmes have gone some way to providing an avenue for support once an adverse event has arisen [29, 30], but crucially, the issue of preparedness has been overlooked. The present study sought to address this by taking a prevention-based approach, aiming to emotionally prepare HCPs before these events occur, in order to reduce their negative impact [31]. We also designed the intervention such that it could be adapted to closely align to a range of healthcare professional disciplines, rather than being limited to any individual group.

The intervention we developed was based on a synergy of adverse event research, psychological resilience research [18] and cognitive-behavioural therapy (CBT) principles [32]. It was theoretically underpinned by the Bi-Dimensional Framework for resilience research [33, 34] which suggests that resilience factors are those which reduce the likelihood of negative outcomes such as distress and depression following exposure to risk factors such as stressful events. Research using this framework has suggested that psychological flexibility, higher self-esteem and a more positive attributional style confer resilience to the negative psychological effects of failure events [18]. As such, the intervention aimed to develop these psychological factors using CBT techniques, alongside providing practical resources and information regarding coping with adverse events. The intervention, which is discussed in more details in the methods section, involved a half-day group workshop and subsequent one-to-one coaching phone call with the facilitator which together provided opportunities for both group exercises and individualised reflective discussions.

One pertinent question regarding the delivery of resilience training interventions relates to the optimal timing of delivery in terms of healthcare professionals’ career pathways. Two key arguments suggest that these should be delivered early in the training pathway, as part of the curriculum. First, newly-qualified healthcare professionals are vulnerable to the psychological impact of stressful healthcare events and there is a need to prepare them before an event arises [35, 36]. Second, delivering interventions during pre-qualification training programmes enables access to large numbers of future clinicians; this may be the most effective way to reach the ‘critical mass’ needed for culture change [37]. However, it is also possible that these groups may be less able to relate to these events and could be less likely to engage with such an intervention. In order to address this, the present study recruited both student and qualified healthcare professional groups and investigated whether feasibility differed between the two groups.

## Methods

### Research aims

The main aim of the present study was to conduct a mixed-methods evaluation of a resilience coaching intervention designed to enhance healthcare professional and student preparedness for involvement in stressful healthcare events, particularly adverse events. A secondary aim was to investigate the issue of timing of delivery by comparing feasibility between student groups who received the intervention as part of their curriculum and qualified professional groups.

### Design

The study used an uncontrolled before-after design which evaluated a resilience training intervention which aimed to enhance participants’ preparedness for involvement in subsequent stressful workplace events. Data were collected at four time points: prior to the intervention (Time 1), immediately following the workshop (Time 2), immediately following the coaching phone call (10–20 days after the workshop; Time 3) and 4–6 weeks after the workshop (Time 4; see Table 1). Outcome measures were informed by the Kirkpatrick model [38] for assessing training interventions. This model suggests that evaluations should collect data at four levels:

1. Reactions/opinions regarding the intervention
2. Learning - increases in knowledge/skills
3. Behaviour - the extent to which learning is applied
4. Results - whether the intervention produces organisational improvements

**Table 1:** Data collection time-points

	Time 1	Time 2	Time 3	Time 4
When:	Prior to workshop	Immediately after the workshop	10–20 days post- workshop	4–6 weeks post- workshop
Alongside event:	Not applicable (online)	Workshop	Coaching phone call	Follow up evaluation phone call
Researcher:	Not applicable (online)	Lead workshop facilitator	Lead workshop facilitator	Independent qualitative researcher
What:	Knowledge assessment	Knowledge assessment		
	Confidence questionnaire	Confidence questionnaire	Confidence questionnaire	Confidence questionnaire
		Feedback/Reactions to the training questionnaire		
	Brief resilience questionnaire		Brief resilience questionnaire	Brief resilience questionnaire

For Level 1, we collected feedback regarding perceptions of the intervention immediately following the workshop using a questionnaire. We also undertook qualitative interviews with a random subset of participants to explore their perceptions of the intervention. For Level 2, participants completed a knowledge assessment which tested knowledge about resilience and coping strategies. While we did not collect data directly pertaining to Level 3 (behaviour), for Level 4, we conceptualised staff confidence and perceived resilience as an organisational improvement, as mental wellness of the healthcare workforce is regarded as an organisational quality indicator [39, 40]. The primary outcome measures were confidence in coping with adverse events (Level 4) and the knowledge questionnaire (Level 2). The secondary outcome measure was self-perceived resilience as measured by the Brief Resilience Scale (Level 4) [41]. Authors JJ, RS-E, LA and LB collected, managed, analysed and reported the quantitative data. Authors GJ, TM and RH collected, managed, analysed and reported the qualitative data. Once analysis of both data sets was complete JJ and GJ met to discuss synergies between the quantitative and qualitative findings and JJ checked the qualitative themes for face validity.

We adopted a mixed-methods approach for complementarity reasons, reflecting the ‘levels’ at which Kirkpatrick recommends collecting data when evaluating training interventions. Combining a brief individual feedback questionnaire after the workshop with a qualitative interview after the full intervention permitted a more comprehensive examination of participant responsiveness (Level 1) and also allowed us to identify any inter-method divergence [42]. Quantitative datasets were particularly important in being able to determine changes in levels of knowledge and mental wellness after the intervention (Kirkpatrick’s levels 2 and 4).

### Setting

Workshops were delivered in locations suitable to each group, including National Health Service (NHS) trust sites and on university premises. The two phone calls following the intervention were received by participants in a location suitable for them.

### Eligibility

Workshops were delivered to groups of single-discipline healthcare professionals or students including midwives, doctors, paramedics, physician associate students and sonography and mammography students. As such, the eligibility criteria for participating in each workshop was being a health professional within that discipline or completing an education programme leading to a particular qualification.

### Intervention

We describe the intervention in line with TIDIER checklist guidance [43]. The resilience training intervention comprised a 3.5 h group workshop and 1 h one-to-one coaching phone call with a facilitator. The facilitators (RS-E/JJ) had a background in CBT-based interventions. JJ is a Clinical Psychologist who has published articles and books on CBT and who has delivered CBT-focused teaching and training. RS-E is an Occupational Health Psychologist who has extensive experience of delivering CBT-based group interventions and one-to-one coaching in occupational and organisational contexts. To support fidelity to the intervention, an intervention protocol was developed together by the facilitators and 6 of 9 workshops were co-facilitated by both facilitators to enable ongoing observation and feedback between the facilitators regarding adherence to the protocol. Of the remaining three workshops, two were facilitated by JJ and one was facilitated by RS-E. Both facilitators also met regularly to discuss the content and process of the coaching phone call to support protocol adherence in this part of the intervention. However, fidelity was not formally measured or assessed.

#### Workshop

The workshop was delivered to discipline specific groups of 4–12 healthcare professionals or students. It was hosted either at the university where the lead author is based or the workplace where participants were based, depending upon what was preferable to each group. The workshop content was underpinned by an evidence-based concept of resilience to failure events, which suggests that individuals who are higher on mental flexibility (or lower on perfectionistic rigidity), higher on self-esteem and have a more positive attributional style are better able to cope with these events (Table 2) [18]. Resilience to failure events was chosen as adverse events can be regarded as a type of failure event, where failures are usually observed to have occurred at a range of levels within the healthcare system. As such, those psychological factors which confer resilience to other forms of failure could be postulated to also confer resilience to adverse events. The workshop drew on the cognitive-behavioural model to identify evidence-based techniques for developing these traits and abilities [32]. These techniques were communicated via psycho-educational didactic teaching, small group discussion and experiential exercises. Each workshop used a series of workplace case studies. These were tailored to the stressful work events commonly experienced by each disciplinary group, with a particular emphasis on adverse events. Workshops also provided information regarding practical and hospital trust/region-specific information resources for coping in the aftermath of adverse events.

**Table 2:** Intervention components

Goal	Targets	Materials	Techniques
More flexible thinking	Normalise stress and failure experience; increase awareness of negative thinking habits, interactions between behaviour, mood and cognition, understanding of interactions between physiology and cognition - towards personal action plan	Video case studies; worksheets; tailored case studies; videos of techniques such as breathing exercises	Experiential exercises; group discussion, reflection; psychoeducation; individual exercises and homework in preparation for coaching phase
Higher self-esteem	Increasing awareness of self-esteem and its impacts; increasing personal self-esteem when experiencing stress and feelings of failure	Worksheets of esteem exercises; self-affirmations; information	Experiential exercise, group discussion and individual exercises and homework in preparation for coaching phase
Better explanatory style	Increasing understanding of and impact of explanatory styles in the context of stress and failure; increasing ability to identify and correct personal habits	Tailored case study	Didactic session, group discussion and reflection, individual exercises and homework in preparation for coaching phase

#### Coaching phone call

The one-to-one phone call with a facilitator aimed to provide a forum for participants to relate the material in the workshop to their own experience. It expanded on three areas which were introduced within the workshop: the development of coping strategies; the affirmation of core values and developing understanding of core strengths. It also provided an opportunity for participants to ask questions or explore issues which they did not feel comfortable discussing in a group setting. Participants could accept this phone call in a location of their choice. During the intervention, it became apparent that the coaching phone calls could vary in length. To manage this, during the intervention the phone calls were modified to limit them to 1.5 h and participants were provided with a warning of the impending end of the phone call at 1 h and 15 min.

### Recruitment and procedure

We used a purposive sampling method to recruit participants from a range of healthcare disciplines and also to sample both student and qualified groups. While we aimed to recruit from a range of healthcare disciplines, it was not possible to recruit from all key healthcare groups due to lack of access to participants (e.g., nurses). Workshops were organised by key contacts within organisations or training programmes and were conducted between November 2018 and June 2019 in Northern England. These contacts circulated the information sheet, eligibility criteria and details of the day and location of the workshop. Once each workshop had been organised, a link to the online baseline questionnaire (Time 1) was circulated. Participants who did not respond to the online survey questionnaire prior to attending were asked to complete a paper copy of the questionnaire before the start of the workshop. Participants completed a paper questionnaire and provided feedback data at the end of the workshop (Time 2). They were asked to respond to a further survey via phone after the coaching phone call (10–20 days after the workshop; Time 3), and a final survey 4–6 weeks after the workshop with an independent researcher whom they had not met before (Time 4; see Table 1). At Time 4, four participants from each group were randomly selected to participate in a qualitative interview. Where groups only comprised four participants all four were invited to the telephone interview. When we were unable to collect data from participants at a specific time point, we still contacted them at the following time point. For example, participants who did not participate in the coaching phone call (Time 3) were still contacted at Time 4 to participate in the survey and interview, where relevant.

### Measures

To evaluate feasibility of implementing the intervention and gathering relevant outcome measures, we recorded participant retention at each time point following baseline. We collected demographic data regarding participant occupational group, age, gender and ethnicity. The primary outcome measures were confidence in coping with adverse events and a knowledge assessment. As no suitable scale or assessment existed, these were created for the purposes of the study. The Confidence in Coping with Adverse Events Questionnaire ('*Confidence'*) contained three items, for example “If I was involved in an adverse event for which I thought I held some responsibility I know the things I would do to help manage my stress levels”. Items were marked on a 4-point scale (from ‘No – not at all’ to ‘Yes – definitely), creating a total possible score range of 3–12. The knowledge assessment ('*Knowledge*') included 3 multiple choice questions and 2 free text responses. It aimed to measure knowledge communicated within the workshop, including information about resilience factors, coping strategies and self-knowledge (personal strengths). Total possible scores ranged from 0 to 6, as one free-text question had a total possible score of 2.

The secondary outcome measure was the Brief Resilience Scale ('*Resilience*') [41]. This contains 6 items which measure perceptions of personal resilience, including ‘I tend to bounce back quickly after hard times’. Items were marked on a 5-point scale (from ‘Strongly disagree’ to ‘Strongly agree’), creating a total possible score range of 6–24. The scale has been found to have a test-retest reliability of .69 over 1 month and to converge with responses to longer resilience questionnaires [41].

We collected feedback data immediately following the workshop (Time 2). Four items were scored on a 5-point scale (from ‘Strongly disagree’ to ‘Strongly agree’). These included ‘The workshop was relevant to my professional group’, ‘I learned skills in the workshop which will be useful in the future’, ‘There was adequate time to cover the material’, and ‘I found the workshop engaging’. A further four items offered ‘yes/no’ response options, and the opportunity to expand on these yes/no responses using free text. These included ‘Were there any aspects of the workshop you did not find useful?’, ‘Is there anything else you would have liked to see in the training which was not included?’, ‘If you were involved in an adverse event, would you do anything differently as a result of attending this workshop?’ and ‘Would you recommend the training to other healthcare professionals?’

### Quantitative data analysis approach

Participants were compared on whether they dropped out at each time-point by their ages and disciplines. For categorical outcomes, comparisons were made using Chi-squared tests. For continuous outcomes, Welch’s *t*-tests were used.

To investigate potential effectiveness, we compared scores on the outcome measures of *confidence*, *knowledge* and *resilience* between baseline (Time 1) and post-intervention time-points (Times 2, 3 and 4). We employed simple random intercepts linear mixed models (restricted maximum likelihood estimation) through the R *lme4* package [44]. This choice of approach was made in part as it allowed us to minimize loss of power. We were aware that our limited sample size and numerous follow-ups may have reduced statistical power to detect effects and mixed models have one key advantage over the more commonly used repeated measures ANOVA; data are retained for each participant up-to the point at which they dropped out. Analyses were also repeated in STATA using the *xtmixed* function while specifying the *vce(robust)* option (calling Huber-White sandwich estimation). There was no evidence of divergence between analyses using standard and robust variance estimation [45, 46], so standards models were presented.

As well as random effects (intercepts) for participants, models included fixed effects of time-only in unadjusted models, and time, gender and age in adjusted models. Where there was one pre-intervention session and three follow-ups, the basic unadjusted model took the form:

$$ {y}_{ij}={\beta}_0+\beta {T}_1x\  Time{1}_{ij}+\beta {T}_2x\  Time{2}_{ij}+\beta {T}_3x\  Time{3}_{ij}+{u}_j+{\varepsilon}_{ij} $$

Where *y*_*ij*_ is the outcome, in participants (_*j*_) with multiple responses (_*i*_), and *βT*_*x*_*x Timex*_*ij*_ represents a dummy exposure variable with pre-intervention = 0, and the relevant timepoint = 1. *u*_*j*_ is the individual subject level error (with variance ${\sigma}_u^2$), *ε*_*ij*_ is the cluster level error (with variance ${\sigma}_e^2$). Clustering was determined using the intraclass correlation coefficient (ICC): the ratio between between-cluster variance and total variance (i.e. between + within-cluster variance):

$$ \mathrm{ICC}=\frac{\sigma_u^2}{\sigma_u^2+{\sigma}_e^2} $$

Where ICC ≥ 0 represents the percentage of variation in the dependent variable explained by between-subject differences over within-subject differences. Model fit was determined using Akaike’s Information Criterion (AIC) (where lower values indicate better fit between adjusted/unadjusted models) and pseudo-*R*^2^ where *R*^2^_M_ indicated the percentage of variance explained by the model fixed effects over total variance and *R*^2^_C_ the fixed *and* random effects over the total variance [47].

Where relevant, post-hoc tests were conducted to compare between time-point means. All tests were adjusted using the Sidak method to mitigate Type I error inflation. Comparisons are presented with *Cohen’s d* estimates (calculated as the mean of the difference between timepoint scores across participants, divided by the standard deviation of the difference between scores). Throughout, *α* was set at .05.

### Qualitative data analysis approach

We collected qualitative feedback data via interviews with a randomly selected sample of participants from each uni-disciplinary cohort to ensure all professional groups attending the training were included. Interviews were conducted and analysed by independent qualitative researchers with backgrounds in nursing, qualitative health research and psychology respectively (GJ; TM; RH) who were not involved in delivering the intervention. Qualitative data collection explored i) participants’ perceptions of the concept of resilience in healthcare and ii) what they thought worked well and what did not work well, to establish ways in which the intervention may be improved (see Additional File 1 for Topic Guide). Data pertaining to the latter question only is reported here.

Interviews were transcribed verbatim. The initial analysis was completed by two researchers with backgrounds in nursing and psychology respectively (GJ; RH). Each researcher listened to the audio recordings repeatedly to become familiar with the data and then independently conducted line-by-line coding of the transcripts. Through line-by-line coding, the researchers took an inductive approach to derive key concepts and phrases regarding experiences and perceptions of the intervention [48]. Through a series of discussions between the two researchers, initial themes were developed from the coding [49]. Refinement of themes and subthemes evolved over subsequent discussions through the course of the analysis until full agreement was reached on the final themes. A third researcher with a background in clinical psychology (JJ) then assessed the themes for face validity [50].

## Results

### Participant characteristics

We delivered uni-disciplinary workshops to Midwives (2 groups; *n* = 19; 28.8%), Paramedics (1 group; *n* = 5; 7.6%), Obstetric and Gynaecology trainee doctors (1 group; *n* = 5; 7.6%), Paediatric trainee doctors (1 group; *n* = 4; 6.1%), Paediatric consultant doctors (1 group; *n* = 4; 6.1%), Physician Associate students (2 groups, *n* = 18; 27.3%) and Sonography and Mammography students (1 group, *n* = 11; 16.7%). Altogether 3 groups (*n* = 29; 43.9%) were delivered to healthcare professional students, and 6 groups (*n* = 37; 56.1%) were delivered to qualified healthcare professionals. 53 (84.1%) participants were female and participants had a mean age of 35.4 (SD = 11.3). 49 (80.3%) participants were White, with remaining participants (*n* = 17; 19.7%) from a range of Black and Asian minority ethnic groups (‘British Indian’ (*n* = 2), ‘British Pakistani’ (*n* = 1), ‘Chinese’ (*n* = 1), ‘Indian’ (*n* = 1), ‘Middle East’ (*n* = 1), ‘Pakistani’ (*n* = 1), ‘Asian’ (*n* = 1), ‘Black British’ (*n* = 1)).

### Retention

We recruited a total of 66 participants to 9 intervention workshops. We retained 62 (93.9%) participants at Time 2, 47 (71.2%) participants at Time 3, and 33 (50%) participants at Time 4. There were no age differences in participants who dropped out at Times 2 and 3, though there was a large significant difference at Time 4; those who dropped out were much younger (Cohen’s *d* = − 0.68, *p* = .01). A higher drop-out in the student groups than the qualified groups likely explains this effect. First, there was evidence of a discipline retention effect where Sonography and Mammography students had the highest dropout at Times 2 (*n* = 4/11, 34%, Cramér’s *ϕ* = 0.57, *p* = .03) and 3 (*n* = 7/11, 64%, Cramér’s *ϕ* = 0.47, *p* = .01). At Time 4, Physician Associate students also had a high dropout rate (*n* = 13/18, 72%). Overall, the student groups had higher drop-out odds than the qualified groups at Time 2 (OR = 12.5, *p* = .03), Time 3 (OR = 3.03, *p* = .05) and Time 4 (OR = 5.55, *p* < .001).

### Primary analyses

**Fig. 1:**
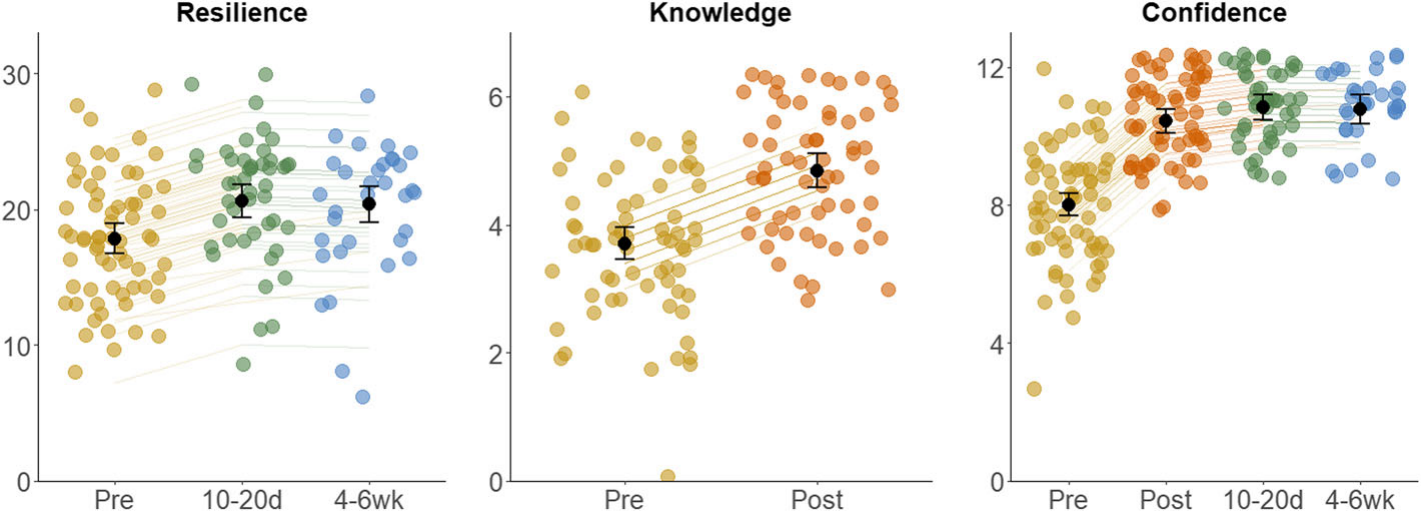
Model fit and results for the outcomes of resilience, knowledge and confidence

Descriptive statistics are presented in Table 3. Model fit and results are presented in Table 4 and Fig. 1. All analyses indicated considerable clustering, supporting the use of random intercepts. The proportion of variance explained by an indicator variable of timepoint was generally substantial across outcomes (with the exception of *resilience* scores, *R*^*2*^_*M*_ = 8%) though variance explained was much higher when factoring in the fixed timepoint effect plus random effects. The inclusion of additional covariates of gender and age across adjusted models did not meaningfully alter model fit.

**Table 3:** Descriptive statistics

Measure	Timepoint	*n*	Mean (*s*)	Missing *n*
Confidence	*Time 1*	66	8.02 (1.69)	0
	*Time 2*	61	10.46 (1.23)	5
	*Time 3*	46	10.85 (0.99)	20
	*Time 4*	33	10.85 (0.97)	33
Knowledge	*Time 1*	66	3.71 (1.12)	0
	*Time 2*	61	4.89 (0.95)	5
Resilience	*Time 1*	65	17.88 (4.64)	1
	*Time 3*	46	20.93 (4.45)	20
	*Time 4*	33	19.88 (4.84)	33

**Table 4:** Model results and fit between outcomes and adjusted and unadjusted models

Outcome	AIC	Variance	*R*^*2*^_*M*_	*R*^*2*^_*C*_	Predictor	Contrast	*β*	95% CI
Unadjusted models								
Confidence *n* of obs*.* = 206	673	${\sigma}_u^2$ = 0.72	47%	69%	Time	T2 v T1	2.43	2.08–2.79
		${\sigma}_e^2$ = 1.03				T3 v T1	2.81	2.42–3.21
		ICC = 0.41				T3 v T1	2.75	2.31–3.19
Knowledge *n* of obs*.* = 127	374	${\sigma}_u^2$ = 0.27	23%	42%	Time	T2 v T1	1.14	0.82–1.46
		${\sigma}_e^2$ = 0.83						
		ICC = 0.24						
Resilience *n* of obs*.* = 144	788	${\sigma}_u^2$ = 15	8%	75%	Time	T3 v T1	2.77	1.82–3.73
		${\sigma}_e^2$ = 5.73				T4 v T1	2.54	1.45–3.62
		ICC = 0.72						
Models adjusted for age/gender								
Confidence adj. *n* of obs*.* = 203	663	${\sigma}_u^2$ = 0.69	48%	69%	Time	T2 v T1	2.41	2.06–2.77
		${\sigma}_e^2$ = 1.03				T3 v T1	2.79	2.40–3.19
		ICC = 0.40				T4 v T1	2.72	2.27–3.16
					Age	*–*	0.01	−0.01-0.04
					Gender	M v F	−0.52	−1.20-0.17
Knowledge adj. *n* of obs*.* = 124	365	${\sigma}_u^2$ = 0.26	23%	41%	Time	T2 v T1	1.09	0.77–1.41
		${\sigma}_e^2$ = 0.81			Age	*–*	0.01	−0.01-0.03
		ICC = 0.24			Gender	M v F	0.00	−0.56-0.55
Resilience adj. *n* of obs*.* = 142	775	${\sigma}_u^2$ = 14.2	14%	75%	Time	T3 v T1	2.76	1.81–3.71
		${\sigma}_e^2$ = 5.71				T4 v T1	2.57	1.49–3.66
		ICC = 0.71			Age	–	−0.09	−0.18-0.00
					Gender	M vs. F	2.20	−0.60-5.01

*Notes*. Likelihood ratio tests for all random effects *p* < .001

*Confidence* scores at each post-intervention timepoint (Times 2, 3 and 4) were significantly higher than pre-intervention (Time 1) scores: Time 2 (unadj. *β  =* 2.43, 95% CI 2.08–2.79, *p* < .001; adj. *β  =* 2.41, 95% CI 2.06–2.77, *d* = 1.55, *p* < .001), Time 3 (unadj. *β  =* 2.81, 95% CI 2.42–3.21, *p* < .001; adj. *β  =* 2.79, 95% CI 2.40–3.19, *d* = 1.71, *p* < .001) and Time 4 (unadj. *β  =* 2.75, 95% CI 2.31–3.19, *p* < .001; adj. *β  =* 2.72, 95% CI 2.27–3.16, *d* = 1.52, *p* < .001). Post-hoc tests indicated that there were no other significant time-point differences (all ps > .17): Time 4 versus Time 2 (e.g. adj. mean diff. = 0.30, 95% CI: − 0.29-0.90, *d* = 0.27, *p* = .37) and Time 3 (e.g. adj. mean diff. = − 0.08, 95% CI: − 0.70-0.54, *d* = − 0.08, *p* = .75); and Time 3 versus Time 2 (adj. mean diff. = 0.38, 95% CI: − 0.15-0.91, *d* = 0.37, *p* = .18). Age and gender effects, as with all subsequent models, were small and non-significant.

*Knowledge* scores were significantly increased At Time 2 versus Time 1 (unadj. *β  =* 1.14, 95% CI 0.82–1.46, *p* < .001; adj. *β  =* 1.09, 95% CI 0.77–1.41, *d* = 0.86, *p* < .001).

For *resilience*, the results were analogous to *confidence*; Time 3 (unadj. *β  =* 2.77, 95% CI 1.82–3.73, *p* < .001; adj. *β  =* 2.76, 95% CI 1.81–3.71, *d* = 0.90, *p* < .001) and Time 4 (unadj. *β  =* 2.54, 95% CI 1.45–3.62, *p* < .001; adj. *β  =* 2.57, 95% CI 1.49–3.66, *d* = 0.65, *p* < .001) scores were both significantly higher than Time 1, though not significantly different from each other (unadj. mean diff. = − 0.24, 95% CI: − 1.60-1.13, *d* = − 0.02, *p* = .68).

### Feedback

Overall, feedback regarding the workshop was positive (Table 5). Most participants agreed or strongly agreed that it was relevant for their professional group; they learned useful skills, it was adequate in length and it was engaging. Of the minority of participants who suggested that there were aspects of the workshop they did not find helpful (9.1%), free text responses were varied, with two participants suggesting that they did not appreciate the introduction (e.g., ‘Was more engaging after the introduction’). Of the minority of participants who would have liked additions to the training (7.6%), three referred to a greater consideration of systems (e.g., ‘cultural impact’, ‘time to discuss how our experiences could be fed back into our organisation’). Most participants said they would do something differently due to the training (83.3%). Free text responses indicated that participants would use different psychological coping strategies (e.g., ‘coping mechanisms and refer to this material’, ‘reframing thoughts, recognising pervasive thoughts, beliefs affecting emotions’), and some also indicated an intended change in practical and behavioural strategies (e.g., ‘I know who to discuss with now’). Most participants also said they would recommend the training to other healthcare professionals (60%) with free text responses underscoring this (e.g., ‘I found this enlightening’, ‘More sessions for staff would be great’).

**Table 5:** Responses to the feedback questionnaire

Item	Strongly disagree (%)	Disagree (%)	Neither agree nor disagree (%)	Agree (%)	Strongly agree (%)	Missing (%)
The workshop was relevant to my professional group	0 (0)	0 (0)	0 (0)	16 (24.2)	46 (69.7)	4 (6.1)
I learned skills in the workshop which will be useful in the future	0 (0)	0 (0)	0 (0)	22 (33.3)	40 (60.6)	4 (6.1)
There was adequate time to cover the material	0 (0)	3 (4.5)	2 (3.0)	30 (45.5)	26 (39.4)	5 (7.6)
I found the workshop engaging	0 (0)	0 (0)	0 (0)	18 (27.3)	44 (66.7)	4 (6.1)
	Yes (%)	No (%)	Missing (%)			
Were there any aspects of the workshop you did not find useful?	6 (9.1)	55 (83.3)	5 (7.6)			
Is there anything else you would have liked to see in the training which was not included?	5 (7.6)	51 (77.3)	10 (15.2)			
If you were involved in an adverse event, would you do anything differently as a result of attending this workshop?	55 (83.3)	4 (6.1)	7 (10.6)			
Would you recommend the training to other healthcare professionals?	60 (90.9)	0 (0)	6 (9.1)			

### Qualitative analysis

Twenty-three interviews were conducted with randomly selected health professionals from all professional groups who took part in the intervention; 18 were female. Of the 23 participants, four were Paediatric Consultant doctors, four were Physician Associate students, four were Midwives, four were Paediatric Trainee doctors, three were Paramedics, three were Sonography or Mammography students and one was an Obstetric and Gynaecological Trainee doctor. Four themes were identified: (1) Tension between mandatory and voluntary delivery; (2) The importance of experience and reference points for learning; (3) Valuing peer learning and engagement; (4) Opportunities to tailor learning.

#### Tension between mandatory and voluntary delivery

Whilst all interviewees identified at minimum that there were some beneficial aspects of the training, the group was divided around whether the training would have the same impact if it was mandatory for staff. Many participants highlighted that voluntary attendance in this study had led to an atmosphere in the room that enhanced the experience of the intervention for participants. Interviews revealed the variety of attitudes across health care staff around preparedness for adverse events and the concept of resilience training. Some felt strongly that resilience should be a mandatory skillset for health professionals and requires basic training, where others raised concerns regarding the implications of mandatory training.

*P1 (Paramedic) - I do feel that erm it should be mandatory because, at least initially in the basic training it should be covered and then further training can be voluntary*

*P2 (Paediatric Trainee doctor)- The challenges of getting time off at such short notice to do it [brief pause] so if it was built into a training programme where people were you know hospitals were forced to give people time off*

*P6 (Paramedic) – I think there’s a danger of, if people were told they had to go on the course I think that would be unhelpful and they would be a bad influence in the room. For those who wanted to do it*

#### The importance of experience and reference points for learning

A number of interviewees highlighted the need to have some experience of the health system and adverse events to draw upon to get the most out of the intervention. Participants who had been in health care for some time were possibly more easily able to reference past events and consider how they might apply the knowledge and skills gained.

P1 *(Paramedic)*– *[having extensive health service experience] helped me and it I hope it helped er the other attendees*

P3 *(Midwife)* – *I related it to me, related it to real life you know as I said, all the case studies that we discussed, I’ve been through I’ve done it you know so I think the majority of the sort of midwives there would’ve been through one of, you know, one of the same adverse incidents*

#### Valuing peer learning and engagement

Participants converged on the value of a small group structure in the initial workshop element of the intervention, citing the benefits of stimulating discussion and engagement of all attendees. The mix of didactic and small group content, and the duration of session was positively received.

*P7 (Obstetrics and Gynecology Trainee doctor*) *– It was good that it was a relatively small group it meant that people could be a bit more open…and discuss really…It encourages discussion points doesn’t it?… plenty of time to discuss what was your view…plenty of chance to kind of interact with the facilitator and the other members of the group.*

*P2 (Paediatric Trainee doctor) – Good I actually like the didactic bits because they were broken up, they were very relevant, very practical erm and then the discussion points*

*P3 (Midwife) – I’m really impressed with the sort of, the way the study day was yeah, you know it wasn’t too long… it was split up nicely, we did team work*

*P4 (Sonographer/Mammographer student) – She kept the interest for the time… your kind of concentration can sometimes wander. I didn’t find that that happened because I think the way they broke it down was quite good into sort of sections…*

#### Opportunities to tailor learning

The coaching phone call was critical to the consolidation of knowledge and for attendees to understand how they might apply their new-found skills in their personal context. The phone call component was identified in most interviews as a central and impactful aspect of the intervention. Some participants reported that they did not anticipate the impact the phone call would have on them and that this required careful planning to ensure they chose a suitable location and time for the discussion.

*P1 (Paramedic) – I found the follow-up phone call personally very useful because I learnt things about myself that I hadn’t even considered*

*P2 (Paediatric Trainee doctor) - Yeah that [the phone call] would probably be my only thing so that made me a little uncomfortable afterwards… it was a really helpful discussion, but it was probably more private than the situation I was in*

*P5 (Midwife) – I think the phone call… gives you the opportunity to [brief pause] to have your say on a more personal level*

### Synergies between qualitative and quantitative findings

In several ways, the qualitative data aided understanding and interpretation of the quantitative results. First, the quantitative data indicated a higher drop-out rate in younger participants and in the student groups than the qualified groups. This converged with the qualitative theme, “Importance of Experience and Reference Points for Learning”, which highlighted how experience of working in healthcare and being involved in adverse events helped participants to engage better with the intervention. Second, the quantitative data indicated that participants’ confidence and resilience levels increased after the workshop and were maintained following the coaching phone call. The qualitative interview comments indicated that this may be explained by the complementarity of these approaches: where participants appreciated the opportunity to engage with their peers in the workshop (reflected in the theme “Valuing Peer Learning and Engagement”) they then subsequently benefited from the opportunity to tailor the learning to themselves in a confidential setting (reflected in the theme “Opportunities to Tailor Learning”).Third, the quantitative data indicated that most participants found the workshop to be ‘engaging’. The qualitative theme ‘Valuing Peer Learning and Engagement’ helped explain why participants may have found this, suggesting that this was a result of the benefits of stimulating discussions with peers and a varied use of exercises.

There was also some divergence between the qualitative and quantitative findings. The feedback sheet responses indicated that most participants would recommend the training to others. However, the interviews revealed that there were caveats or tensions in this regard. The theme, “Tension between Mandatory and Voluntary Delivery” suggested that whilst participants felt this training was crucially important, it would fail if all staff were informed they must attend. Similarly, the theme, “Importance of Experience and Reference Points for Learning”, indicated that participants considered that the timing of offering this intervention was crucial, and more experienced clinicians would be able to engage better.

## Discussion

This paper reports a mixed-methods evaluation of a resilience training intervention designed to enhance healthcare professional and trainee preparedness for involvement in stressful healthcare events, particularly adverse events, and to explore the potential effectiveness of the intervention. Data were collected at four time points: baseline, immediately after the workshop element of the intervention, immediately after the coaching phone call and four-to-six weeks after baseline. Retention was high for the intervention components, with the large majority of participants completing the workshop, the coaching phone call and the questionnaires which were collected immediately after these. Drop-out was higher for the final data collection time-point however, with half of participants not responding to this. Retention was higher in qualified staff than students.

The outcome data indicated that participants reported higher confidence in coping with adverse events, greater relevant knowledge and higher levels of general resilience after the intervention. This gain was maintained over each post-baseline time-point. The feedback data for the workshop were overwhelmingly positive; all participants agreed or strongly agreed that the training was engaging and relevant to their discipline and said they had learned useful skills. The qualitative analysis identified four themes. The first addressed the ‘tension between mandatory and voluntary delivery’, suggesting that resilience is a mandatory skillset but identifying drawbacks to making the intervention a mandatory training requirement. The second focused on the ‘importance of experience and reference points for learning’, with many participants suggesting the intervention was more appropriate for qualified staff than students. The third focused on the workshop, considering how participants valued ‘peer learning and engagement’. This suggested that participants felt the small-group nature of the workshops and mixture of activities maximised benefits. The fourth addressed the coaching phone call, suggesting this provided valuable ‘opportunities to tailor learning’, but highlighting the need to prepare participants for the personal nature of this.

These findings extend the literature in three main ways. First, these results support the feasibility of delivering a psychological resilience intervention to multidisciplinary HCPs and provide preliminary evidence that this may be effective for improving practitioners’ confidence in coping with adverse events and enhancing their general resilience. These findings are consistent with the broader literature on resilience interventions, which suggests that these are generally acceptable and effective for increasing levels of resilience across populations and occupational groups [51, 52]. However, only a limited literature has previously tested resilience interventions in HCPs and these have usually focused on single-disciplinary healthcare groups [22–24, 27, 28]. Furthermore, commentators have expressed concerns about the use of these in healthcare contexts [6, 25, 26]. We considered these criticisms carefully in the design of this intervention, which led us to focus specifically on the stressful events which can be considered an intrinsic demand of healthcare occupations, not amenable to organisational interventions. Our results extend the existing literature by suggesting that such a tailored intervention is broadly acceptable and that it is possible to create one intervention which can be easily adapted to enhance resilience in a range of different healthcare disciplines. As such, our findings suggest that this intervention should be further investigated using a more robust, controlled study design. These findings are timely given the additional stressors healthcare professionals are currently experiencing due to Covid-19.

It should be noted, however, that the final data-collection time point which occurred outside of an intervention component showed poorer retention, challenging the feasibility of collecting data in this way in a scaled-up evaluation of the intervention. One possibility to enhance retention might be to incentivise participants for completing the questionnaires. Alternatively, feasibility may be enhanced if scaled-up evaluations focus on the recruitment of qualified staff rather than students, as retention was significantly higher at all post-baseline time points in qualified participants. The qualitative findings complemented this by suggesting that this was because the qualified staff may have been better able to relate to topics being discussed during the intervention. This finding also addressed one of our research questions regarding which point during health professionals’ career pathways the intervention should be delivered. While it could be argued that delivering the intervention as pre-qualification, as part of the standard curriculum, may provide better access to participants and could help prepare students before an adverse event arises, it instead seems that these groups may be less able to relate to these events and as such could be less likely to engage with the intervention. Finally, it must also be considered that retention may be higher if the ‘dose’ of the intervention is increased; the current intervention was only a half-day workshop combined with a single coaching phone call, which is notably shorter than a number of other previously tested resilience interventions which run over multiple full days (e.g., [22, 24, 53]). In order to extend the existing intervention, additional exercises could be included to help develop targeted psychological skills and more time could be taken to complete all exercises. As the feedback item regarding time suggested that some participants felt there was not adequate time to cover the material, this change would also address this finding. It could be hypothesized that by increasing the time participants spend receiving the intervention, the ‘social contract’ to then participate in follow-up surveys and interventions may be enhanced [54]. However, it must also be acknowledged that by increasing the length of the intervention, more participants may become unable to participate in specific sessions due to clinical or personal demands.

Second, this study is the first to investigate whether it may be possible to pro-actively prepare HCPs for involvement in adverse events in particular. This element of our focus was unique as previous resilience interventions in healthcare professionals have focused more broadly on developing resilience to stress and workplace stress generally (e.g., [22–24, 53]). We focused on adverse events as these occur in 10% of hospital admissions [8] and there is now a widespread awareness that these can be psychologically damaging for the HCPs involved [12]. Previous interventions to support healthcare professionals with adverse events have taken a reactive approach, providing support only once adverse events have occurred [29, 30]. The present research extends this literature by providing the first preliminary evidence indicating that HCPs perceive benefits to a pro-active intervention and that this could be useful for increasing their confidence in coping with adverse events and their knowledge around this. However, further research is needed to confirm these findings.

Third, our findings contribute to the debate on whether psychological skills interventions should be mandatory or optional for healthcare staff [55, 56]. At present, there is no mandatory requirement for most qualified healthcare professionals internationally to take part in any such training. This may support individual choice and reduce the risk that participants will perceive that they are being ‘singled out’ or referred to the training due to demonstrating some deficiencies in coping ability [22]. However, it may also increase the risk that such training is ‘pushed out’ in favour of clinical work and participants who might want to take part are unable to do so [55, 56]. Qualitative findings from our study suggested that participants felt psychological resilience is a key skill for any healthcare professional which supports their practice and as such, making it mandatory would be appropriate. Conversely, participants were concerned that due to the group structure of the workshop and the sensitive and personal nature of the topic material, including participants who may not be interested or ready for such training could create a negative atmosphere, harming the experience of others. As such, our findings suggest that while managers should be supportive of staff taking part in such interventions, participation should not be mandatory.

### Strengths and limitations

The study benefited from drawing on a multidisciplinary participant group, which showed that the intervention was acceptable in a range of healthcare contexts. It also benefited from including measurement of outcomes across multiple time points and from the inclusion of an independent qualitative evaluation. However, it was limited by its uncontrolled design which meant that findings cannot be interpreted as evidence of effectiveness. Furthermore, two of the measures were designed for the purposes of the study as no suitable validated questionnaires were available. It was also limited by a lack of fidelity measurement: we did not monitor the coaching phone calls for fidelity to the model and suggest that in future, evaluations of this intervention should do this. Lastly, a large degree of drop-out meant that post-intervention between timepoint comparisons had low power, meaning any subtle longitudinal effects could not be detected.

## Conclusions

There is contention around the appropriateness of delivering resilience interventions in healthcare contexts but the high levels of stress and burnout which are reported by healthcare professionals continue to drive a need for these types of interventions. The current study suggests that a resilience intervention which is focused specifically on the intrinsic challenges of healthcare work and which is tailored to the stressors that different disciplines will encounter is acceptable to participants. It also provides preliminary evidence that it may be effective for enhancing confidence in coping with adverse events, relevant knowledge and more general resilience in these groups.

## Supplementary Information

**Additional file 1.**

## Data Availability

The deidentified datasets used and/or analysed during the current study are available from the corresponding author on reasonable request (email: j.johnson@leeds.ac.uk). The data are not publicly available due to their containing information that could compromise the privacy of research participants.

## Abbreviations

AIC: Akaike’s Information Criterion
CBT: Cognitive behavioural therapy
HCP: Healthcare professional
ICC: Intraclass correlation coefficient
NHS: National Health Service
