# Supplementary material for: Can we prepare healthcare professionals and students for involvement in stressful healthcare events? A mixed-methods evaluation of a resilience training intervention
Source: BMC Health Serv Res. 2020 Nov 27;20:1094. doi: 10.1186/s12913-020-05948-2 (PMC7691965; doi:10.1186/s12913-020-05948-2)
Supplement: Supplementary file 1 — Additional file 1. [file 12913_2020_5948_MOESM1_ESM.docx]

**Additional File 1**

**Topic Guide**

*Outline of the project. Check participants’ understanding of the study information, offer an opportunity for additional questions and go through the consent process.*

**RESILIENCE TRAINING INTERVENTION**

1. What was your overall perception of the resilience training intervention?
2. How has your involvement benefited you?

Prompts:

- understanding of the concept of resilience;
- altered resilience;
- home life/work life;
- ability to cope with instances of error;
- relevant skills for future career

**WORKSHOP and FOLLOW UP phone-call/tutorial**

I’d now like to ask you a bit about you’re your experience of the workshop.

1. What was your overall perception of the workshop?
   1. Probe particular issues arising here
2. What did you think worked well?
3. What could be improved?
   1. Probe as to how improvements might be made

Prompts: length of time, 3 sessions, size of group, balance of theory/practical examples, interactive exercises, relevance to particular staff group, voluntary/mandatory

1. What was your experience of the follow up phone-call/tutorial?
   1. Probe particular issues – 10 day gap; tailored/personal; repetition
2. How did this call contribute to your learning and overall professional development in relation to this topic?
   1. Probe particular issues arising here
3. Overall would you recommend this session to others undertaking your professional training?

*That brings us to the end of the question I have for you but do you have anything you would like to add or any questions for us? Thank the participant.*
